# National Health Insurance Notes

**Published:** 1923-02

**Authors:** 


### NATIONAL HEALTH INSURANCE NOTES.

### THE SALE OF INSURANCE PRACTICES.

THAT Insurance practices are bought and sold, just as private practices change hands, is common knowledge. But all the clamour and excitement of a year or two ago because a provision in the 1920 Regulations was supposed to deprive doctors of the saleable value of their practices was bound to fail in the attempt to get the Regulations amended. No Government could possibly recognise that a doctor had a vested interest in his insurance practice. The doctors, having now accepted this fact, have secured what they were really groping after all the time—a recognition of the fact that the successor to a practice is subject to the risk of being called upon to treat the patients of his predecessor during the period in which they are making up their minds about "choosing a doctor." This generally results in their doing the most obvious thing—going to Dr. B, who has taken over Dr. A's practice. The circular letter of December 21, 1922, issued by the Ministry of Health to Insurance Committees, indicates an approved method by which Dr. B will be suitably paid in respect of this insurance risk. It is solely a question of the distribution of the funds available for the doctors in the Insurance Committee area. No compulsory assignment of insured persons will be made during a period of eighteen months (instead of three as at present) after the death or voluntary resignation of a doctor. During that period every such insured person will be at liberty to choose a doctor, and Dr. B will receive each quarter such extra share of the fund as the Panel Committee and the Insurance Committee may agree, as representing the responsibility for treatment of his predecessor's patients who have not yet chosen a doctor, which he undertook when giving notice, on the death or resignation of Dr. A, that he was willing to accept his patients. The Department make it clear that the initiative in regard to the adoption of an arrangement of this kind must come from the Panel Committee in each area, and that, though it will be approved, it is not in any way pressed on Insurance Committees.

### THE UNWILLING BENEFICIARY.

We confess to a certain sympathy for the individual subscribing himself "£230 a year" who wrote to the daily press on January 1, stating that he was diffident about taking advantage of the Health Insurance benefits, and whose contributions were therefore merely a tax which provided him with no return—a tax from which others of his fellows, with higher salaries, were exempt. But our sympathy would extend equally to anyone who is compelled to contribute for a benefit which he does not want. His request that the income limit should be lowered is no remedy, and his suggestion that persons beyond a certain limit should have the option whether to be insured or not is an impracticable insurance proposition with a uniform rate of contribution, and that is essential. So far as the medical benefit provisions of the Act are concerned, there is quite a false conception abroad about "taking advantage" of the services of the panel doctor. He is just as surely paid by an insurance premium as if he were paid a fee for each attendance. It should not be a difficult matter for any doctor to make this clear to patients who have qualms about "taking advantage" of the panel system to get medical attention. This is not free of charge, but merely paid for in a particular way to which some of the patients are unaccustomed.

### INSURANCE FINANCE.

The "amazing article" to which we referred in our last issue has been so completely disowned by the Editor of "Health," who announces that it has brought him a volume of authoritative condemnation, that one is disarmed from further criticism. We refer to the matter to give the following information in regard to the general distribution of insurance moneys in substitution for the various crazy alternatives which were given in the article in question. Out of every 20s. received in the Insurance Funds for England and Wales, whether from contributions or the Exchequer, or interest on the accumulations in the Funds, a sum of 12s. 2½d. is estimated to be spent on benefits, 5s. 1½d. carried to reserve, and 2s. 8d. spent on administration. This information was given in the House of Commons in July last in answer to a question.

### SWINGS AND ROUNDABOUTS.

In the published report of an interview in which the resolutions of the Annual Panel Conference were brought before the Ministry, we read that complaint was made that persons who had ceased to be entitled to medical benefit were continuing to receive treatment from panel doctors, owing to the lateness of notifications received from the Approved Societies by the Insurance Committees, who are powerless to act until they receive such notifications. One doctor said that if conditions throughout the country were the same as in Manchester the loss to practitioners through the wrongful use of medical cards and the non-notification of suspensions from medical benefit must amount to between £50,000 and £70,000 a year. Sir Walter Kinnear promised to look into cases submitted to him from Manchester and other areas, and added, on a different point, that it might be some consolation to the Committee to know that the medical pool was estimated to be receiving an advantage of about £150,000 a year, owing to the large number of persons who left one society and entered another as new entrants during their free year of insurance, and for whom credits were allowed twice. Thus the deficiency on the swings would appear to be nicely covered by the surplus on the roundabouts. And yet some of the doctors went away feeling that there is a catch somewhere.

---

NURSES' REGISTRATION.—The Minister of Health has given notice, as required by Statute, that the Register of Nurses has now been compiled. The effect of this notice will be that after three months from December 5, 1922, the unauthorised use of the title "Registered Nurse" will render a person liable to a fine not exceeding £10 for a first offence and £50 for any subsequent offence. The first Register is now being printed, and will be published by the General Nursing Council, 12 York Gate, Regent's Park, N.W.1.

